# The Natural and Artificial Methods of Feeding Infants and Young Children

**Published:** 1899-03

**Authors:** 


**The Natural and Artificial Methods of Feeding Infants and Young Children.** By Edmund Cautley, M.D. Pp. viii., 376. London: J. & A. Churchill. 1897.

Of the many works that have been produced during the last few years on this subject there is not one that we can recommend more heartily than this. There is little that is new in it, but the author has condensed into a comparatively small space, without undue curtailment, all the recent investigations on the methods of preparing foods for the infant and juvenile stomach. The chapter on proprietary foods is particularly good, and we are in thorough accord with the author in his dictum that in the vast majority of cases in which they are being alone given the child is being deprived of more suitable and appropriate food. We admit with him that several may with advantage be given on occasions: it is the abuse of them that we condemn, and the abuse is more common than the appreciation.

